# Telework during the COVID-19 epidemic in Portugal and determinants of job satisfaction: a cross-sectional study

**DOI:** 10.1186/s12889-021-12295-2

**Published:** 2021-12-06

**Authors:** Mafalda Sousa-Uva, António Sousa-Uva, Marta Mello e Sampayo, Florentino Serranheira

**Affiliations:** 1 CHRC – Comprehensive Health Research Center; National School of Public Health, NOVA University of Lisbon, Lisbon, Portugal; 2 Department of Epidemiology, National Institute of Health Doutor Ricardo Jorge, Lisbon, Portugal; 3 Department of Occupational and Environmental Health of the National School of Public Health, NOVA University of Lisbon, Lisbon, Portugal; 4 ISCTE- University Institute of Lisbon, Lisbon, Portugal

**Keywords:** Telework, Job satisfaction, Occupational health, Ergonomics, COVID 19 pandemic

## Abstract

**Background:**

Telework satisfaction is a Public Health concern, intensified by the COVID-19 pandemic, and its determinant factors may be related with the negative health effects of teleworking. However, there is still little research exploring this issue. This study aimed to characterize telework during the first wave of the COVID-19 epidemic in Portugal and to identify the major predictors of telework satisfaction.

**Methods:**

This is a cross-sectional study aimed at all teleworkers working in Portugal, during the first wave of the COVID-19 epidemic. Data were collected through a Google Forms platform online questionnaire distributed by a snowball method on social networks. Descriptive statistics included crude and relative frequency data. The associations between sociodemographic characteristics, self-perceived health, organization of working time, concentration at work, work-life balance, work disconnection, working conditions, and organizational demands (flexibility and organizational trust based on E-work Life Scale) with telework satisfaction were estimated through logistic regression.

**Results:**

This study included 1004 participants. Teleworkers satisfaction levels were high (69%). Better concentration at work (OR = 1.54; 95%CI 1.01–2.34); the satisfaction with the balance between work life and extra work when teleworking (OR = 1.79; 95%CI 1.17–2.74); and higher work flexibility (OR = 2.26; 95%CI 1.46–3.49) were good predictors of greater levels of satisfaction with telework. However, its major predictors were the company’s trust in teleworkers (OR = 4.50; 95%CI 2.89–7.02) and feeling good in the workspace at home (OR = 3.72; 95%CI 1.46–9.49).

**Conclusions:**

Our findings point that work environment and organizational culture play a crucial role in affecting telework satisfaction. More studies are needed to monitor telework satisfaction and its effects on physical and mental health, so that Public and Occupational Health (and Safety) can be able to identify and implement the best interventions that allow promoting individual health and foster a healthy work environment for teleworkers.

## Background

Teleworking is a designation that dates back to the 1970’s [1]. It refers to work outside the principles of companies or other organizations, which determines the almost mandatory use of information and communication technologies (ICTs) [2].

Nowadays, it is more and more frequent to observe employees out of their usual offices or workplaces [3]. They work in conference rooms, in clients’ offices, at coffee shops, at home, in their cars, at airports and anywhere there is internet connections. People are working in these places at all times of the day (during usual working hours, at night and on weekends) using a wide variety of ICTs, including, mostly, internet, smartphones, home computers, laptops, tablet computers, teleconferencing, and videoconferencing [4].

During the COVID-19 pandemic, more than 3.4 billion people in 84 countries have become confined to their homes, as estimated in late March 2020, which potentially translates too many millions of workers temporarily exposed to telecommuting [5]. Strict social distancing measures were applied in Portugal, as well as in other European countries, including nationwide lockdown [6]. The first lockdown, during the first wave of the COVID-19 epidemic in Portugal, took place in March 18 and remained until mid and late May. Telework became obligatory in March 19 until early July. Hence, telework was forced.

Telework gave workers the opportunity to work from their homes or elsewhere, with less time spent travelling to work, with higher autonomy and flexible timing [7]. However, findings from research developed before COVID-19 pandemic showed that telework could have both positive and negative effects, depending on teleworkers’ job profiles, on dimensions of job quality measured, on support received from employers, on personal preferences, as well as on family structure [8].

Previously described positive effects of telework include improvement of job satisfaction [8] and work-life balance [9]; and less work-life conflicts [10]. Major negative effects are physical and mental health adverse effects (mental distresses, as stress, anxiety, depression, and physical conditions as work-related musculoskeletal disorders) which can arise related to isolation, limited mobility in home, working conditions, number of working hours and breaks; as well as unbalance between work demands and workers abilities [11–13].

The Occupational Health intervention becomes very difficult in what concerns about telework, by the lack of knowledge of concrete workstations [14]. Thus, this is nowadays a major (and growing) Public and Occupational Health challenge. The working conditions such as lighting, indoor air quality and thermal conditions are frequently diverse and unknown. In addition, work overload (physical and/or mental) can be increased causing potential negative effects on health [15]. The room in which worker develops occupational activities in the household is usually unknown, which is also fundamental for occupational risk management [16]. The psychosocial risks of teleworking are even more difficult to assess. That is because they are not restricted to cognitive, emotional, or mental constraints. Telework can be an occupational hazard for work-related stress and anxiety, depression or even chronicity of some of these health events [17].

For most remote employees, telework came with the COVID-19 pandemic being their first teleworking experience [12]. Parents were apparently forced to support their children during office hours with the closure of schools and many teleworkers didn’t have a private room specifically designed for work, and/or internet connection and/or adequate digital devices to work and/or for children’s’ distance learning.

Studies performed in different countries have been describing the negative effects of telework during the COVID-19 pandemic in physical [18] and mental health outcomes [19]. Telework during COVID-19 crisis was described affecting workers well-being, job satisfaction, work-life balance [17], and also productivity [20].

However, there is still little research concerning this organizational work. The COVID-19 pandemic offered a unique opportunity to investigate telework, as such a large number of workers in teleworking had never been observed in the past. The first step to better understand the possible health effects of telework is to explore which are the major determinant factors of telework satisfaction, as people with worst levels of telework satisfaction should be those more at risk of having the negative effects of it. Thus, this study aimed to characterize telework during the first wave of the COVID-19 epidemic in Portugal and to identify the major predictors of telework satisfaction considering sociodemographic characteristics, self-perceived health, concentration at work, organization of working time, work-life balance, work disconnection, working conditions in an Occupational Health perspective, as well as organizational demands, namely, flexibility and organizational trust.

## Methods

### Data sources and study population

We designed an observational, analytic, cross-sectional study aimed at all teleworkers working in Portugal, during the first wave of the COVID-19 epidemic.

Data were collected through a self-administered online questionnaire built using the Google Forms platform, which was distributed online, by a snowball method on social networks (Linkedin; Facebook; WhatsApp). Thus, this is a convenience sample that reflects the telework satisfaction of those who filled out this questionnaire. All participants were volunteers and were informed about the study purposes. Data collection took place between May 12th and June 3rd, 2020.

Accordingly, an individual self-administered questionnaire was developed to assess the following topics: (i) sociodemographic characteristics of respondents (sex, age group, marital status, education, children at home) and self-rated health, based on a single item from SF-36 scale, measured on a five-point Likert scale (1- very good; 5- very bad) [21]; (ii) telework satisfaction (adapted from work satisfaction [22]), concentration at work, organization of working time, work-life balance and work disconnection, based on single items of E-work life scale [23]; (iii) the equipment used at home (computer, laptop, keyboard, mouse) and working conditions considering current guidelines concerning ergonomic principles that may be applied to the design of dialogues between humans and informatics systems and recommendations of the Japan Human Factors and Ergonomic Society [24–26] and (iv) organizational demands (flexibility and organizational trust dimensions of E-work life scale [23]). E-work life scale is a 17-item scale with four factors: organizational trust, flexibility, work-life interference and productivity [23]. We used two dimensions of the E-Work Life Scale: organizational trust with 3 items and flexibility with 3 items. Answers were given on a five-point Likert scale (1- disagree; 5 - strongly disagree).

### Data analysis

The analysis was restricted to those who self-reported to be in telework in the 4 weeks prior to filling out the questionnaire. Thus, we focused the analysis to individuals that answered “*yes*” to the question “*Are you (or have you been in the last four weeks) telecommuting?”.* From the total number of 1079 participants, 75 were excluded due to this inclusion criterion. The final sample size was then of 1004 participants.

The participants answered to the question “*How satisfied are you with your work today?”* [22]. We grouped “very satisfied” with “satisfied”; and “neither satisfied nor dissatisfied” with “dissatisfied” and with “very dissatisfied” to generate the variable telework satisfaction (yes vs. no).

The English version of the questions considered in the questionnaire were translated into Portuguese language. We followed Brislin’s (1980) translation/back-translation procedure to create a Portuguese version of them [27]. Items were translated to Portuguese by the authors and were then submitted to peers that were fluent in both Portuguese and English. Blind peer back-translation was performed to check item’s consistency and both second and third authors validated the translation process. Concerning to the flexibility and organizational trust dimensions from E-work life scale [23], the description of the variables under study were analyzed, as well as their correlations. Factor analysis was performed for each construct and the internal consistency of the respective items was calculated. Cronbach’s alphas were 0.7 for both organizational trust and flexibility. The dimensions organizational trust and flexibitity from E-work life scale were calculated using factor analysis. To ascertain the necessary assumptions for factor analysis implementation was used the Kaiser–Meyer–Olkin measure of sampling adequacy. All components were rotated using varimax (orthogonal) rotation to maximize factor loadings. Factors were retained based on Kaiser’s Criterion (eigenvalues ≥1). Factor scores were calculated for each individual using Bartlett’s approach. The dimensions were computed by averaging their items and the scores were recoded into 3 categories (high, medium, low).

Descriptive statistics included crude and relative frequency data. Binary logistic regression was applied to estimate the associations between sociodemographic variables, self-perceived health, organization of working time, concentration at work, work disconnection, work-life balance, feeling good in the workspace at home, having support from Health and Safety at Work to adapt furniture and computer equipment, and organizational demands (flexibility and organizational trust), with telework satisfaction. Regression model fit was estimated with the Hosmer-Lemeshow goodness of fit test. To verify if the results were not a consequence of the functional form selected, we used a probit model. The level of significance was fixed at 5%. Statistical analysis was performed in PASW version 20. The statistical confidence level was set at 95%.

## Results

In this study participated 1079 individuals, of which 75% are female, 64% are between 30 and 49 years old, and 60% are married or in a union.

From the total number of participants who are currently in telework or have been in the last 4 weeks (*n* = 1004), 91% are doing telework for more than a month; 76% are women; 39% have between 40 and 49 years old; 60% are married or in a union; and 46% are graduates (Table 1). The health state perception was good, or very good for 73%.

**Table 1:** Characteristics of the respondents who self-reported to be in telework in the four weeks prior to filling out the study questionnaire

Variable	Categories	n	Frequency (%)
**Socio-demographic and self-rated health**			
Sex	Men	245	24.40
	Women	759	75.60
	Missings	0	0.00
Age group	20–29 years	80	7.97
	30–39 years	249	24.80
	40–49 years	393	39.14
	50–59 years	216	21.51
	≥ 60 years	66	6.57
	Missings	0	0.00
Education	Less than High School	76	7.70
	Graduation	459	46.27
	Master degree	340	34.27
	PhD	117	11.79
	Missings	12	1.20
Marital status	Single	279	27.79
	Married or in an union	601	59.86
	Widowed	14	1.39
	Divorced	110	10.96
	Missings	0	0.00
Children’s at home	None	514	51.20
	1 child	211	21.02
	2 children	236	23.51
	3 or more children	43	4.28
	Missings	0	0.00
Self-rated health	Good or very good	732	72.90
	Average	26	2.60
	Bad or very bad	246	24.50
	Missings	0	0.00
**Telework satisfaction, organization of working time, concentration at work, work life balance and work disconnection**			
Telework satisfaction	Yes	690	68.80
	No	313	31.20
	Missings	1	0.10
Intention to perform telework in the future	Yes, permanently	99	9.86
	Yes, in part time	604	60.16
	Yes, sporadically	218	21.71
	No	83	8.27
	Missings	0	0.00
I am satisfied with the balance between work life and extra work when teleworking	Totally agree or agree	534	53.10
	Neither agree nor disagree	100	10.00
	Partially disagree or Strongly disagree	370	36.90
	Missings	0	0.00
I know when I must disconnect work to be able to rest when teleworking	Totally agree or agree	499	49.70
	Neither agree nor disagree	77	7.70
	Partially disagree or Strongly disagree	428	42.70
	Missings	0	0.00
Number of hours teleworking compared to usual	More hours than usual	597	59.46
	Fewer hours than usual	95	9.46
	Identical to the number of hours previously worked	312	31.08
	Missings	0	0.00
Establishment of a working time during teleworking	Always	466	46.40
	Sometimes	411	40.90
	Rarely	127	12.60
	Missings	0	0.00
Taking breaks when working with the computer	I don’t take breaks	49	4.90
	I take short breaks sporadically	631	62.80
	I take several regular breaks	324	32.30
	Missings	0	0.00
I can concentrate better when teleworking	Totally agree or agree	531	52.90
	Neither agree nor disagree	210	20.90
	Partially disagree or Strongly disagree	263	26.20
	Missings	0	0.00
**Organizational work demands**			
I feel that the work demands are much greater when teleworking	Totally agree or agree	576	57.30
	Neither agree nor disagree	199	19.80
	Partially disagree or Strongly disagree	229	22.80
	Missings	0	0.00
**Flexibility**			
My work is so flexible I could easily take time off e-working remotely, if and when I want to	Totally agree or agree	315	31.40
	Neither agree nor disagree	156	15.50
	Partially disagree or Strongly disagree	533	53.10
	Missings	0	0.00
My supervisor gives me total control over when and how i get my work completed when e-working	Totally agree or agree	165	16.40
	Neither agree nor disagree	115	11.50
	Partially disagree or Strongly disagree	724	72.10
	Missings	0	0.00
My line manager allows me to flex my hours to meet my needs, providing all the work is completed	Totally agree or agree	215	21.40
	Neither agree nor disagree	138	13.70
	Partially disagree or Strongly disagree	651	64.80
	Missings	0	0.00
**Organizational trust**			
My organization trusts me to be effective in my role when e-work remotely	Totally agree or agree	756	75.30
	Neither agree nor disagree	145	14.40
	Partially disagree or Strongly disagree	103	10.30
	Missings	0	0.00
I trust my organization to provide good e-working facilities to allow me to e-work effectively	Totally agree or agree	551	54.90
	Neither agree nor disagree	144	14.30
	Partially disagree or Strongly disagree	309	30.80
	Missings	0	0.00
My organization provides training in e-working skills and behaviours	Totally agree or agree	342	34.10
	Neither agree nor disagree	217	21.60
	Partially disagree or Strongly disagree	445	44.30
	Missings	0	0.00
**Equipment used at home and working conditions**			
Equipment used in telework	Computer (tower) and peripherals	94	9.40
	Laptop without peripherals	457	45.50
	Laptop computer with peripherals	437	43.50
	Other (tablet; mobile phone; ...)	16	1.60
	Missings	0	0.00
Mousepad (mouse interface panel on the laptop) use	Always	204	44.70
	Sometimes	114	25.00
	Rarely	138	30.30
	Missings	1	0.22
How the top of the laptop monitor look when sitting in relation to the horizontal	Above eye height	23	5.10
	At eye level	141	31.00
	Below eye height	291	64.00
	Missings	2	0.44
How the top of the computer monitor look when sitting in relation to the horizontal	Above elbow height	74	17.9
	At the same height as the elbow	218	51.9
	Below elbow height	128	30.5
	Missings	111	20.90
Height of the external keyboard when using periphericals	Above elbow height	109	28.70
	At the same height as the elbow	255	67.10
	Below elbow height	16	4.20
	Missings	151	28.44
My company support internet payment	Yes	43	4.30
	No	961	95.70
	Missings	0	0.00
I feel good in my workspace at home	Always	625	62.30
	Sometimes	346	34.50
	Rarely	33	3.30
	Missings	0	0.00
Lighting in the workplace at home where you are teleworking	Adequate	822	86.80
	Inadequate	125	13.20
	Missings	57	5.70
Health and Safety at Work gave support on how to adapt furniture and computer equipment	Yes	246	24.50
	No	758	75.50
	Missings	0	0.00

The majority of respondents felt satisfied and very satisfied with telework (69%) and would like to do it in the future (92%), but mostly in part-time (60%). Respondents doing telework were also satisfied with the balance between work and their life (53%). Some have difficulties to disconnect from work to rest (50%) and 60% consider that “work more hours than usual”. Furthermore, 46% “always” establish a working time and 63% take sporadic and short breaks when working with the computer. The majority of respondents can concentrate better when teleworking (53%) (Table 1).

Considering organizational demands, data reveals that: 57% of respondents felt that work demands are much greater when teleworking*;* 53% partially disagree or strongly disagree that work is so flexible that can easily take a break if/or when workers want to*;* and 75% felt organizational trust on their performance. Regarding resources, 55% of teleworkers felt that the company *gives all the conditions and resources* to do work at home, and mostly (66%) did not had enough assistance and training to develop skills to do work at home (Table 1).

Results show that 46% use a laptop without peripherals (external monitor, keyboard, and mouse). The interface panel (mousepad) is used in 45% of these cases and 69% works with the laptop below or above the eye height. In those who use peripherals, working positions outside comfort angles related to keyboard position were observed, 33% works with the keyboard below or above the height of the elbows. Concerning to the monitor position, 48% of the respondents work with the top of the monitor below or above eye height (Table 1).

Companies had provided frequently desk computers and laptops but did not supported internet payment (96%). Most respondents enjoy their workspace at home (62%), considering the illumination as good (87%). When asked about “the existence of someone from the company in the Health and Safety at Work area, who gave support on how to adapt furniture and computer equipment”*,* data reveal that 76% of respondents had no support whatsoever (Table 1).

Results from logistic regression revealed that sociodemographic variables weren’t associated with telework satisfaction, including having children at home. Good or very good self-reported states of health were associated with higher odds of being satisfied with telework (OR = 2.32; 95%CI 1.63–3.30) (Table 2).

**Table 2:** Results from logistic regression to identify the major factors associated with telework satisfaction (yes vs. no)

Independent variables	Independent variables categories	***p*** value	Adjusted Odds Ratio	Adjusted Odds Ratio 95%CI	
				Lower	Upper
Sex	Men	0.73	0.94	0.39	1.37
	Women^a^				
Age group	20–29 years	0.78	0.88	0.36	2.16
	30–39 years	0.23	0.64	0.31	1.33
	40–49 years	0.69	0.86	0.42	1.79
	50–59 years	0.85	0.93	0.45	1.92
	≥ 60 years^a^				
Education	Less than High School	0.18	0.59	0.28	1.28
	Graduation	0.13	0.66	0.39	1.13
	Master degree	0.45	0.81	0.46	1.41
	PhD^a^				
Marital status	Single	0.41	0.76	0.40	1.46
	Married or in an union	0.97	0.77	0.44	1.36
	Widowed	0.96	1.04	0.25	4.26
	Divorced^a^				
Children’s at home	None	0.73	0.87	0.38	1.97
	1 child	0.45	1.39	0.59	3.25
	2 children	0.77	0.88	0.39	2.02
	3 or more children^a^				
Self-rated health	Good or very good	< 0.001	2.32	1.63	3.30
	Average, bad or very bad^a^				
I am satisfied with the balance between work life and extra work when teleworking	Totally agree or agree	0.01	1.79	1.17	2.74
	Neither agree nor disagree	0.61	0.87	0.50	1.50
	Partially disagree or Strongly disagree^a^				
I know when I must disconnect work to be able to rest when teleworking	Totally agree or agree	0.08	0.69	0.45	1.04
	Neither agree nor disagree	0.17	0.65	0.35	1.20
	Partially disagree or Strongly disagree^a^				
Number of hours teleworking compared to usual	More hours than usual	0.07	1.46	0.97	2.22
	Fewer hours than usual	0.36	0.76	0.43	1.36
	Identical to the number of hours previously worked^a^				
Establishment of a working time during teleworking	Always	0.15	1.49	0.87	2.54
	Sometimes	0.67	1.12	0.67	1.85
	Rarely^a^				
Taking breaks when working with the computer	I take short breaks sporadically	0.28	1.49	0.72	3.08
	I take several regular breaks	0.61	1.22	0.56	2.65
	I don’t take breaks^a^				
I can concentrate better when teleworking	Totally agree or agree	0.04	1.54	1.01	2.34
	Neither agree nor disagree	0.63	0.89	0.56	1.41
	Partially disagree or Strongly disagree^a^				
I feel that the work demands are much greater when teleworking	Totally agree or agree	0.06	1.53	0.98	2.38
	Neither agree nor disagree	0.41	0.83	0.53	1.29
	Partially disagree or Strongly disagree^a^				
Flexibility	High	< 0.001	2.26	1.46	3.49
	Medium	< 0.001	2.25	1.45	3.48
	Low^a^				
Organizational trust	High	< 0.001	4.50	2.89	7.02
	Medium	< 0.001	2.18	1.44	3.31
	Low^a^				
I feel good in my workspace at home	Always	0.01	3.72	1.46	9.49
	Sometimes	0.09	2.25	0.89	5.70
	Rarely^a^				
Health and Safety at Work gave support on how to adapt furniture and computer equipment	Yes	0.25	1.26	0.85	1.86
	No^a^				

^a^Reference class

The satisfaction with the balance between work life and extra work when teleworking (OR = 1.79; 95%CI 1.17–2.74); better concentration when teleworking (OR = 1.54; 95%CI 1.01–2.34); higher work flexibility (OR = 2.26; 95%CI 1.46–3.49) and feeling good in the workspace at home (OR = 3.72; 95%CI 1.46–9.49) were also associated with better satisfaction levels with telework (Table 2).

In addition, higher organizational trust (OR = 4.50; 95%CI 2.89–7.02) predicted a greater telework satisfaction (Table 2).

On the other hand, knowing when have to disconnect from work to be able to rest; the establishment of a working time; taking working breaks; feeling that work demands are much greater when teleworking; and having support from Health and Safety at Work to adapt furniture and computer equipment were not associated with telework satisfaction.

## Discussion

In this study, we used logistic regression to investigate the major predictors of telework satisfaction during the first wave of the COVID-19 epidemic in Portugal. Our main results were that work environment and organizational culture play an important role in affecting telework satisfaction.

From the total number of participants who were in telework or have been in the last 4 weeks, during the data collection period (*n* = 1004), the large majority were in telework for more than a month (91%). This means that they have been telecommuting since the period of the state of emergency in Portugal due to the COVID-19 epidemic (about 4 weeks before the questionnaire was completed).

In the exceptional situation that characterized the data collection phase of this study (COVID-19 state of emergency), it was observed that employees have a high level of satisfaction with telework (69%) in comparison with other studies performed during the pre-pandemic phase, although working from home has been commonly associated with job satisfaction [10, 28, 29]. Nevertheless, is in line with research performed during the COVID-19 pandemic [30]. This result may be due to sample presenting higher levels of education, and an eventual higher financial situation, which may mean better working conditions at home, overestimating the levels of satisfaction; as well it may be influenced by feeling safe from COVID-19 at home.

Despite telework satisfaction has been more valued by women than men [31, 32], as telework allows women to plan their work and family time [33], we didn’t find significant associations between sociodemographic factors and the levels of satisfaction with telework. This may be due to telework obligation for both women and men that came with the COVID-19 pandemic. Women more frequently feel that family demands interfere with work activities and the pandemic circumstances may have reinforced gender patterns in the division of domestic tasks and childcare [29].

Previous studies have pointed that teleworkers with children rate their own satisfaction and family well-being higher than those with no children at home [34, 35]. However, in our study, teleworkers with children did not presented significantly higher levels of satisfaction with telework. Such difference with other studies may be related to the different political, economic and cultural contexts, as well as the way COVID-19 risk management was performed by National Health Authorities, and how the population perceived it. Besides, teleworkers were forced to be telecommuting together with children’s at home, having the schools closed and performing distance learning, sometimes without home conditions for doing that, all at the same time, in the same place, but without any chance for choosing [36].

Job demands and resources are described to be the major features that can influence telework satisfaction [37] and previous studies revealed a number of multifaceted implications and advantages of teleworking for individuals, organizations and society [38, 39]. Teleworking is usually associated with a decrease in work-life conflict and improved productivity [9, 40]. Our findings are in agree with that as the concentration at telework (OR = 1.54; 95%CI 1.01–2.34); the satisfaction with the balance between work life and extra work when teleworking (OR = 1.79; 95%CI 1.17–2.74); and feeling good in the workspace at home (OR = 3.72; 95%CI 1.46–9.49) were good predictors of higher levels of satisfaction with telework.

The autonomy and flexibility are also known to contribute for job satisfaction [10, 41, 42] and our results are in line with that as telework satisfaction increased with higher flexibility (OR = 2.26; 95%CI 1.46–3.49) and better organizational trust (OR = 4.50; 95%CI 2.89–7.02).

Our study revealed that teleworkers use laptops frequently without peripherals (45%). The majority use the monitors below or above eye level height (69% use laptops below or above eye level height and 48% computers). These are well-described occupational hazards for Work-Related Musculoskeletal Disorders (WRMSDs). Office workers worldwide commonly report Musculoskeletal disorders (MSDs), being known for its detrimental effects on workers’ health and productivity [43]. Current guidelines regarding monitor placement at Visual Display Users (VDU) suggest that the top of the screen should be at, or slightly below, eye level [25]. Therefore, our results point that telework conditions deserve better attention from Occupational Health perspective.

The strengths of this study are the following: (i) the high sample size to characterize telework and investigate predictors of telework satisfaction; (ii) the fact that there is still little research concerning telework and telework satisfaction; (iii) the study of telework during a period of lockdown of the first wave of the COVID-19 epidemic in Portugal, when telework was mandatory, offering a unique opportunity to investigate this organizational work; and iv) the use of several items and validation for the Portuguese language of flexibility and organizational trust dimensions of the very recent E-Work Life Scale and its application to study telework during the COVID-19 pandemic.

Are considered as major limitations of this study the following: (i) the non-representative sample of the Portuguese population of teleworkers during the first wave of COVID-19 epidemic in Portugal, either in number or in terms of the characterization of respondents; (ii) the selection bias due to sample presenting higher levels of education and an eventual higher financial situation, which may mean better working conditions at home (space, equipment and an improved work environment at home), which may overestimate the levels of satisfaction; (iii) the information bias due to self-reporting; (iv) and results not representing causal associations due to potential omitted variable bias and the cross sectional study design.

Finally, it should be mentioned that although studies performed before COVID-19 pandemic evidence advantages of the implementation of teleworking, is still not clear, under the actual circumstances, the relationship between teleworking and job satisfaction. Actually, we can’t fully apply what had known in the past to the exceptional situation of a pandemic (e.g. with telework forced and schools closed). Therefore, it is important to perform more studies concerning such issue, namely, prospective studies in a representative sample of the population of teleworkers, to monitor telework satisfaction during and after the pandemic period. Teleworking, being a frequent work modality in the future, raises major questions about the best way to protect the health of those that work at home. Therefore, it is important that Public and Occupational Health (and Safety) can be able to identify and implement the best interventions that allow promoting individual health and foster a healthy work environment for teleworkers.

## Conclusions

In this study, workers satisfaction levels with telework were high. Its major predictors were having higher organizational trust and feeling good in the workspace at home. Thus, our study, point that organizational culture and work environment play a crucial role in affecting telework satisfaction.

More research on the determinants of teleworkers satisfaction and well-being is necessary for understanding the role of teleworking on workers’ mental and physical health.

## Data Availability

The datasets used and/or analyzed during the current study are available from the corresponding author on reasonable request.
